# Differential enrichment of bacteria and phages in the vaginal microbiomes in PCOS and obesity: shotgun sequencing analysis

**DOI:** 10.3389/frmbi.2023.1229723

**Published:** 2024-03-26

**Authors:** Senlin Zheng, Huimin Chen, Hongyi Yang, Xulan Zheng, Tengwei Fu, Xiaoyan Qiu, Meiqin Wang

**Affiliations:** ^1^ Third Institute of Oceanography, Ministry of Natural Resources, Xiamen, China; ^2^ Department of Gynecology and Obstetrics, The First Affiliated Hospital of Xiamen University, Xiamen, China; ^3^ Department of Psychology, University of British Columbia, Vancouver, BC, Canada; ^4^ Yanxuan Biotech Ltd., Xiamen, China

**Keywords:** vaginal microbiome, polycystic ovary syndrome, obesity, bacteria, phages

## Abstract

**Introduction:**

Previous research has linked vaginal bacteria to polycystic ovary syndrome (PCOS) and obesity in women, yet the specific disparities in vaginal microbiota between these conditions remain unclear.

**Methods:**

In this study, we aimed to elucidate the contribution of dysregulated vaginal microbiota to PCOS and obesity by analyzing the vaginal microbiota in reproductive-aged women with and without PCOS, as well as obese and non-obese women, using shotgun sequencing.

**Results:**

Swab specimens were collected from four groups of subjects: PCOS and obese, PCOS and non-obese, non-PCOS and obese, and non-PCOS and non-obese. A total of 333 bacteria and 24 viruses/phages were identified to the species level. Clustering analysis revealed that non-PCOS and non-obese individuals exhibit a similar “healthy” vaginal microbiome, while both obesity and PCOS were associated with microbial dysbiosis. Significant differences in abundance were observed for 26 bacterial species and 6 phages/viruses between groups. Notably, pathobionts such as *Streptococcus pyogenes*, *Leptospira santarosai*, *Citrobacter amalonaticus*, *Listeria ivanovii*, and *Clostridium perfringens* were significantly less abundant or absent in the non-PCOS and non-obese group. Furthermore, the abundance of *Lactobacillus*, *Pseudomonas* bacteria, and their corresponding phages exhibited positive correlations. *Lactobacillus* bacteria, *lactobacillus* phage, and *pseudomonas* phage/virus were identified as indicators of a healthy vaginal microbiome. Importantly, the differentially enriched bacteria in the PCOS and obesity groups were distinct.

**Discussion:**

This study confirms that PCOS and obesity are associated with differing enrichment of bacteria and viruses/phages, with both conditions linked to microbial dysbiosis. Moreover, our findings suggest that vaginal phage diversity is associated with a healthy vaginal microbiota, while dysbiosis is associated with a decrease in phages alongside increased bacterial diversity.

## 1 Introduction

Polycystic ovary syndrome (PCOS) is an endocrine disorder affecting women of reproductive age with a worldwide prevalence of approximately 8–13% (Jobira et al., 2020). In patients with PCOS, numerous small cysts (fluid-filled sacs) form in the ovaries. Typical clinical features include abnormally high levels of androgens, irregular menstrual cycles, hirsutism, acne, obesity, and infertility (Jobira et al., 2020).

PCOS pathogenesis remains elusive, but emerging research highlights the role of human microbiome in its occurrence and progression (Duan et al., 2021). Studies have shown that PCOS is associated with dysbiosis of the gut microbiota (Lindheim et al., 2017; Liu et al., 2017; Insenser et al., 2018; Torres et al., 2018; Qi et al., 2019; Jobira et al., 2020; Sun et al., 2023). Patients with PCOS have reduced diversity and altered composition of the gut microbiota, such as a decrease in *Lactobacillus* and *Bifidobacterium*, and an impaired intestinal mucosal barrier, compared to those without any health problems. The alterations in the gut microbiota have been linked to levels of inflammation and insulin resistance, by altering the stability of the intestinal mucosa and metabolites in patients with PCOS (Sun et al., 2023).

There are four pathogenic mechanisms of PCOS: hyperandrogenism (HY), insulin resistance (IR), folliculogenesis dysfunction (FC), and neuroendocrine axis dysfunction (ND) (Hao et al., 2016; Crespo et al., 2018; Torres et al., 2018; He and Li, 2020). Hyperandrogenism is a key contributor to PCOS pathogenesis (Witchel et al., 2022). Previous studies have shown that hyperandrogenism is associated with the gut microbiota (Torres et al., 2019). Androgen affects the gut microbiota, including a decrease in the diversity of the gut microbiota and an enrichment of some bacteria. For example, dihydrotestosterone induces an increase in *Chlamydia* but a decrease in *Escherichia coli* in PCOS mice induced by dihydrotestosterone (Zhang et al., 2019); female rats exposed to high levels of androgens at birth have reduced gut microbiota diversity and an increased risk of metabolic disorders in their adult offspring (Sun et al., 2023). On the other hand, gut microbiota affect testosterone secretion (Torres et al., 2018); for example, transplantation of gut microbiota from male to female mice resulted in abnormal basal metabolism and elevated testosterone levels in the latter (Zhang et al., 2019; Chu et al., 2022). Insulin resistance is thought to be the key metabolic abnormality in patients with PCOS (Sun et al., 2023). Dysfunction of the gut microbiota may lead to a “leaky gut”, leading to increased presence of LPS in the blood and resulting in chronic inflammation (Hao et al., 2016; He and Li, 2020; Zeng et al., 2020). The gut bacteria-induced inflammation, in turn, fosters insulin resistance, obesity, autoimmune diseases, and PCOS in women of reproductive age (Lindheim et al., 2017; Liu et al., 2017; Insenser et al., 2018). It’s noteworthy that insulin can directly stimulate androgen production, hence, hyperinsulinemia in PCOS can concurrently provoke hyperandrogenism (Crespo et al., 2018). Folliculogenesis dysfunction is indirectly linked with gut microbiota dysbiosis. Dysfunction of gut microbiota could lead to hyperandrogenism and insulin resistance, which contribute to the development of PCOS. Excess androgen can block the pathway that converts androgen to estrogens by inhibiting the FSH-stimulated aromatase activity in granulosa cells. Low estrogen levels result in follicular atresia (Zeng et al., 2020). In addition, patients with PCOS have elevated levels of anti-Müllerian hormone (AMH), leading to increased follicular resistance to FSH action. This resistance to FSH action inhibits follicle maturation and ovulation. This leads to the formation of large numbers of immature follicles in the ovary (Crespo et al., 2018; Zeng et al., 2020). Neuroendocrine axis dysfunction (ND) mechanisms contributed to PCOS in some patients. In some PCOS patients, there is resistance to the negative feedback loop of progesterone and GnRH. As the inhibition of progesterone on GnRH production is weakened, more GnRH release from the hypothalamus will lead to excessive LH release from the pituitary and ultimately affect sex hormone production in the ovary and lead to PCOS (Crespo et al., 2018).

The microbiota of the skin and vaginal tract also play a significant role in influencing immune response and inflammation (Huttenhower et al., 2012). In women of reproductive age, a healthy vaginal microbiome typically exhibits a low pH (4.2 to 5.0) in different ethnic groups, with a limited presence of dominant facultative anaerobic bacteria, such as *Lactobacillus crispatus* and *L. inner*, which help maintain an acidic environment and produce H_2_O_2_ as a defense against pathogen invasion (Greenbaum et al., 2019; Hong et al., 2020; Wright, 2021). H_2_O_2_-producing *Lactobacilli* colonize the vagina at rates of up to 10^6^–10^7^ cells/mL.

A earlier study indicated that the diversity of vaginal bacterial is more pronounced in PCOS patients than in the control group. Notably, PCOS patients demonstrated a lower prevalence of species such as *Lactobacillus crispatus, Mycoplasma, and Prevotella* (Hong et al., 2020). However, it is important to note that the authors also highlighted the necessity for further research to explore the causal relationships between PCOS and the composition of the vaginal microbiome. Dysbiosis of vaginal microbiome is associated with the change of vaginal mucosa (Chen et al., 2021; Miko and Barakonyi, 2023). The vaginal mucosa is a critical component of the female reproductive system. This mucosal lining is a complex structure that serves several essential functions, including providing a barrier against infections, facilitating reproductive processes, and contributing to the overall health of the vaginal environment. The vaginal mucosa comprises various cell types and components, including epithelial cells, macrophages, natural killer (NK) cells, dendritic cells (DC), and neutrophils, T lymphocytes, B cells and immunoglobulin IgG and IgA (Chen et al., 2021; Miko and Barakonyi, 2023). The change of vaginal microbiome will not only result a shift of the microbe components, but also linked with the immune responses of the host.

In addition to bacteria, phages are important components of the human microbiome, and they are thought to play a crucial role in shaping microbial composition, driving bacterial diversity and facilitating horizontal gene transfer. Research on the role of phages in the human microbiome and disease remains relatively limited (Zhang et al., 2023). Major studies have focused on the prevalence of phages in the gut of individuals affected by diseases such as ulcerative colitis and diabetes, with a particular focus on exploring the potential relevance of phages to disease. These investigations have shown a correlation between higher numbers of phages in the gut of individuals with ulcerative colitis and diabetes. However, to date, there is no definitive link between the widespread presence of phages in the human body and the dynamics of human health and disease. Notably, phages are distributed in various parts of the human body, and reports on viruses/phages in the vaginal microbiome are still being reported. Further studies are needed to further understand whether there are any significant changes in phage/virus in the vaginal bacteria of PCOS patients and their associated roles (Łusiak-Szelachowska et al., 2020).

The vaginal microbiome has a direct connection to a woman’s reproductive system, which can both influence and be influenced by ovarian health. To date, no study of the vaginal microbiome has used shotgun sequencing to examine the more detailed microorganisms (e.g., phages) in addition to bacteria. The entire microbiome should and does play an important role in the regulation of the vaginal mucosa in patients with PCOS and obesity. Therefore, we performed deeper shotgun sequencing than the 16s test to obtain more specific microbiome information and to explore disease-related factors.

## 2 Materials and methods

### 2.1 Participants

According to the inclusion criteria, PCOS patients admitted to the First Affiliated Hospital of Xiamen University in Fujian Province from June 2018 to June 2019, and healthy volunteers were recruited. Meanwhile, healthy volunteers were recruited for the non-PCOS group. All participants signed an informed consent form before the experiment. All participants underwent a general physical examination and an ultrasonic-B scan before enrollment (**Supplementary Table 1**).

### 2.2 Diagnostic criteria

All patients with PCOS fulfilled the Rotterdam 2003 diagnostic criteria:

(1) Sparse ovulation or anovulation.
(2) Hyperandrogenic manifestations or hyperandrogenemia.
(3) Ovarian polycystic changes (12 or more follicles 2 ~ 9mm and ovarian volume ≥10mL in one or both ovaries).
(4) Patients meeting 2 of the above 3 criteria but with hyperandrogenism caused by other diseases (congenital adrenal hyperplasia, Cushing’s syndrome, ovarian or adrenal tumor) were excluded.
(5) Individuals with a BMI > 28 were considered obese.

### 2.3 Inclusion criteria

(1) All participants with PCOS were women aged 18–40 years who fulfilled the Rotterdam diagnostic criteria.
(2) The control group consisted of 18–40 year old women with regular menstruation and no clinical signs of hyperandrogenism.

### 2.4 Exclusion criteria

(1) Adolescents who did not have regular menstrual periods.
(2) Individuals with inflammatory bowel disease.
(3) Individuals with thyroid dysfunction, hyperlactatemia, congenital adrenal hyperplasia, Cushing’s syndrome, ovarian or adrenal tumors, and other diseases of the endocrine system.
(4) Patients with other chronic diseases, such as tumors, may require chemotherapy, radiation therapy, or long-term oral medications.
(5) Oral or intravenous use of antibiotics or probiotics within the last three months and during the study.
(6) Vegetarian or irregular eater.
(7) Smokers.
(8) Circumstances that the investigator deems unsuitable for participation in the study.

### 2.5 Sample collection and shotgun library preparation

According to BMI and the PCOS diagnosis results, the 41 volunteers were divided into four groups, 11 individuals in the PCOS with obesity (PO) group, eight individuals in the non-PCOS with obesity (NO) group, 8 individuals in the PCOS and non-obese (PS) group, and 14 individuals in the non-PCOS and non-obese (NS) control group. Microbiota samples were collected from the posterior fornix of the vagina were collected using a clean and sterile cotton swab during the non-menstrual period (Greenbaum et al., 2019). Swabs were placed into collection tubes containing fixative (YXA, Xiamen, China) and then delivered to the laboratory in dry ice boxes and stored in a −80 °C refrigerator until nucleic acid extraction. Total DNA from the swaps was then extracted using the DNeasy PowerSoil Kit (Qiagen, Hilden, Germany), as recommended by the Human Microbiome Project. The DNA libraries for NGS sequencing were prepared using the Tn5 tagmentation-based protocol (Hennig et al., 2018). DNA segmentation and addition adaptors simultaneously with the Tn5 enzyme were performed according to Hennig’s protocol (Hennig et al., 2018). The purified DNA libraries were then sequenced on the Illumina HiSeq X sequencing platform using a pair-end (PE150) strategy.

### 2.6 Analysis of sequencing data and microbiota profiling

The raw shotgun sequencing data were used for taxonomic analysis using MetaPhlAn3 (Metagenomic Phylogenetic Analysis, Version 3.1.0) (Beghini et al., 2021) and Kraken2 (Wood et al., 2019). The retrieved microbiota analyzed included bacteria, viruses, and fungi. The function of the microbiota was analyzed using Humann3 (Beghini et al., 2021), followed by megahit assembly (Dinghua et al., 2015).

### 2.7 Statistical methods

The one-way ANOVA test was used to test the significance of the difference in abundance of specific species between groups. The correlation between the bacteria and phage/virus species was analyzed using the R package Hmisc and plotted using the R package pheatmap. The differentially enriched microbial species in the PCOS and obesity groups were analyzed using LEfSe tool (Segata et al., 2011). The significance of the difference in relative bacterial abundance between PCOS and non-PCOS was analyzed with a t-test. The PCA analysis of the vaginal microbiome in samples from different groups was performed using the R package DESeq2 and plotted using ggplot2.

## 3 Results

### 3.1 NGS sequencing results

A total of 41 PE150 sequencing data sets were obtained, and each sample received at least 20 million reads. The data have been uploaded to Sequence Read Archive (SRA) database (Accession No. SRR24561304 to SRR24561344).

### 3.2 Microbiota profiling

#### 3.2.1 Bacteria

The microbiome profile shows 4 phyla (*Actinobacteria, Firmicutes, Proteobacteria*, and *Cyanobacteria*), 4 classes, 9 orders, 11 families, 265 genera, and 333 species of bacteria. There are 220 bacterial species that were observed in only one of the 41 samples, and 33 bacterial species were identified in at least 10 samples. There are 31 genera detected in more than 10 samples, including *Staphylococcus, Lactobacillus, Clostridium, Klebsiella, Xanthomonas, Komagataeibacter, Enterobacter, Pseudomonas, Burkholderia, Streptococcus, Streptomyces, Leptospira, Neisseria, Azospira, Polynucleobacter, Citrobacter, Listeria, Bacillus, Rhodococcus, Pseudonocardia, Sinorhizobium, Corynebacterium, Cupriavidus, Ralstonia, Campylobacter, Acinetobacter, Stenotrophomonas, Enterococcus, Photorhabdus, Cohaesibacter, and Zobellia*. Among them, *Zobellia*. *Lactobacillus, Staphylococcus*, and *Clostridium* were detected in all the samples.

**Figure 1:**
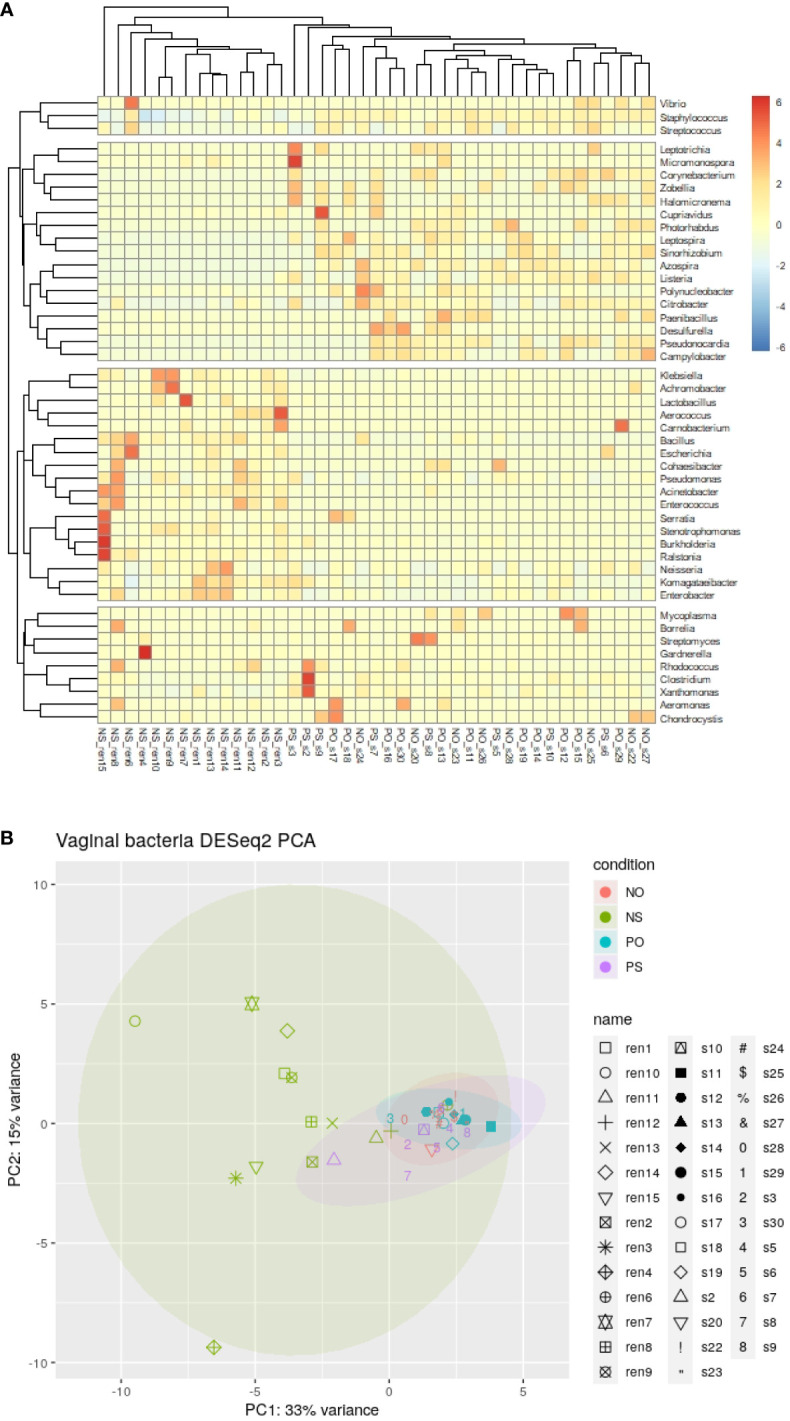
The clustering and PCA results of bacteria genera of the 41 vaginal microbiomes. **(A)** Cluster of bacteria genera; **(B)** DESeq2 PCA.

The clustering result of the samples and bacterial genera shows that all samples of the NS group clustered in the same clade, as shown in the heat map (**Figure 1A**). In contrast, the other 3 groups (PO, PS, and NO) clustered in different clades (**Figures 1A, B**). This result suggests that only the non-PCOS and non-obese group have normal vaginal microbiome, with low diversity and bacterial species of *Lactobacillus*. Thus, obesity and PCOS may lead to dysbiosis of the vaginal microbiome. Bacterial genera were grouped into four clades (**Figure 1A**). Lactobacillus is in the same clade as 17 other genera, such as *Klebsiella, Acinetobacter, Pseudomonas*, and *Enterococcus*. *Clostridium* is clustered with 8 other genera, including *Streptomyces* and *Gardnerella*, which are often pathogenic. *Staphylococcus* is clustered with *Streptococcus* and *Vibrio*. The fourth clade contains 17 genera, including *Listeria*, *Leptospira*, *Photorhabdus*, and *Azospira*.

There were 21 bacterial genera with significantly different abundances between groups (ANOVA-test p < 0.05). Ten genera, *Lactobacillus, Klebsiella, Komagataeibacter, Enterobacter, Pseudomonas, Bacillus, Ralstonia, Acinetobacter, Stenotrophomonas, and Enterococcus*, showed higher abundance in the NS group. In comparison, another 11 genera, *Staphylococcus, Clostridium, Leptospira, Azospira, Citrobacter, Listeria, Pseudonocardia, Sinorhizobium, Corynebacterium, Campylobacter*, and *Photorhabdus* were relatively low or not detected in the NS group (**Supplementary Figure 1**).

The results of the ANOVA test showed that there were 26 bacterial species and 6 phages/viruses with different abundance between the groups (**Supplementary Figures 1**, **2**). In the NS group, 16 species show lower abundance than in other groups, including 5 pathogens and 4 opportunistic pathogens. The 5 pathogens included *Streptococcus pyogenes, Leptospira santarosai, Citrobacter amalonaticus, Listeria ivanovii*, and *Clostridium perfringens*. The 4 opportunistic pathogens included *Staphylococcus aureus, Ralstonia insidiosa, Corynebacterium diphtheriae*, and *Photorhabdus asymbiotica*.

The bacterial species detected in no less than 9 out of 41 specimens are listed in **Table 1**.

**Table 1:** The frequently detected bacterial species and their frequency in the PCOS group and in the non-PCOS group.

Name	#Specimens detected out of 41	Frequency (%)			PCOS vs Non-PCOS	
		Mean	Min	Max	Ratio (PCOS/Non-PCOS)	t.test p-value
*Staphylococcus aureus*▲	41	55.17	7.68	81.37	1.36	0.0008
*Komagataeibacter rhaeticus*	40	8.12	0	20.26	0.84	0.16
*Klebsiella michiganensis* *	37	4.6	0	44.53	0.26	0.03
*Klebsiella pneumoniae*▲ ***	38	4.13	0	14.72	0.34	0.0002
*Lactobacillus crispatus* *	27	2.29	0	43.32	0.08	0.04
*Xanthomonas citri* *	39	1.87	0	9.09	1.88	0.01
*Enterobacter cloacae*♦ *	38	1.83	0	6.24	0.55	0.01
*Streptomyces* sp. *ICC1*	31	1.75	0	21.51	1.05	0.47
*Gardnerella vaginalis*▲	9	1.72	0	67.6	0.02	0.16
*Burkholderia dolosa*▲	37	1.53	0	20.79	0.51	0.15
*Pseudomonas fluorescens*▲	38	1.44	0	4.57	1.02	0.47
*Leptospira santarosai*♦ **	32	1.17	0	4.63	2.61	0.002
*Azospira oryzae* *	26	1.05	0	4.44	1.97	0.02
*Clostridium botulinum*♦	38	1.04	0	13.64	2.08	0.14
*Streptococcus pyogenes*♦	34	0.62	0	1.84	1.45	0.08
*Polynucleobacter necessaries*	25	0.56	0	4.44	1.85	0.12
*Lactobacillus johnsonii*	26	0.46	0	4.55	0.89	0.41
*Xanthomonas euvesicatoria* *	22	0.39	0	4.55	3.3	0.04
*Pseudomonas aeruginosa*▲	20	0.35	0	3.18	0.45	0.09
*Rhodococcus opacus*	16	0.31	0	4.55	3.65	0.09
*Lactobacillus delbrueckii* *	28	0.3	0	1.52	2.14	0.01
*Pseudomonas* sp. *PONIH3*	21	0.29	0	1.98	0.87	0.38
*Ralstonia insidiosa*▲ *	14	0.28	0	4.95	0	0.02
*Lactobacillus gasseri*	19	0.27	0	2.73	1.22	0.36
*Listeria ivanovii*▲ *	20	0.27	0	1.11	2.07	0.02
*Citrobacter amalonaticus*♦ *	21	0.24	0	1.11	1.98	0.03
*Lactobacillus amylovorus*	13	0.23	0	2.69	0.54	0.19
*Streptomyces* sp. *ICC4*	16	0.23	0	1.14	1.13	0.4
*Sinorhizobium* sp. *RAC02* **	16	0.22	0	0.83	4.11	0.001
*Lactobacillus jensenii* *	10	0.21	0	3.33	0	0.02
*Bacillus cereus* ***	16	0.2	0	1.52	0.1	0.0008
*Corynebacterium diphtheriae*♦ *	14	0.2	0	0.98	3.6	0.01
*Clostridium perfringens*♦	15	0.18	0	1.11	1.97	0.08
*Clostridium* sp. *DL-VIII* **	16	0.18	0	0.76	3.99	0.001
*Pseudonocardia dioxanivorans* *	16	0.16	0	0.62	2.15	0.04
*Cupriavidus oxalaticus*	15	0.15	0	1.64	2.48	0.09
*cyanobacterium endosymbiont of Epithemia turgida* *	12	0.12	0	0.6	3.5	0.01
*Ralstonia pickettii* *	11	0.12	0	1.98	0	0.02
*Zobellia galactanivorans* **	10	0.12	0	0.76	5.59	0.008
*Photorhabdus asymbiotica*♦	11	0.11	0	0.69	1.22	0.36
*Enterococcus casseliflavus*	11	0.1	0	1.02	0	0.007
*Halomicronema hongdechloris*	9	0.1	0	0.76	3.08	0.05
*Escherichia coli*	9	0.07	0	0.98	0.24	0.07
*Acinetobacter bereziniae*▲ *	10	0.06	0	0.99	0	0.02
*Cohaesibacter* sp. *ES.047*	10	0.06	0	0.51	0.8	0.37
*Stenotrophomonas maltophilia* *	11	0.06	0	0.99	0	0.01

♦ pathogens; ▲ opportunistic pathogens; * p<0.05; ** p<0.01; *** p<0.001.

The clustered matrix displaying the Pearson correlation between detected phages/viruses and bacterial species is depicted. Lactobacillus bacteria and Lactobacillus phage are positively correlated. Most of the *Lactobacillus* species and *Pseudomonas* species are highly correlated. A total number of 26 *Lactobacillus* species were identified, including *Lactobacillus crispatus, L. iners, L. acetotolerans, L. acidipiscis, L. acidophilus, L. alimentarius, L. amylolyticus, L. amylophilus, L. amylovorus, L. apis, L. crustorum, L. delbrueckii, L. gallinarum, L. gasseri, L. heilongjiangensis, L. helveticus, L. jensenii, L. johnsonii, L. kefiranofaciens, L. kullabergensis, L. paracollinoides, L. plantarum, L. reuteri, L. salivarius, L. sanfranciscensis*, and *L. sp wkB8*. Most of them are significantly positively correlated (p<0.01) (**Supplementary File 1**). *Lactobacillus phage.Lv.1* positively correlated with *Lactobacillus iners* (r = 0.41, p = 0.0068), *L. crustorum* (r = 0.43, p = 0.0049), *L. plantarum* (r = 0.44, p = 0.0033), *L. jensenii* (r = 0.52, p = 0.00048), and *L. amylolyticus* (r = 0.58, p = 7.0E-05). There are 68 species of bacteria and 12 species of viruses/phages that are positively correlated with the 26 *Lactobacillus* species, and only one species, *Staphylococcus aureus*, is negatively correlated with *Lactobacillus* species (**Supplementary File 1**).

*Pseudomonas* bacteria and *Pseudomonas* phages/viruses are positively correlated (**Supplementary File 1**). A total of 9 *Pseudomonas* species and 5 *Pseudomonas* viruses/phages were identified, including *Pseudomonas aeruginosa, P. balearica, P. fluorescens, P. pohangensis, P. putida, P. resinovorans, P. rhodesiae, P. stutzeri, and P. syringae;* and *Pseudomonas virus EL, Pseudomonas virus phiCTX, Pseudomonas virus LKA1, Pseudomonas phage PA16*, and *Pseudomonas phage F10*. *Pseudomonas phage F10* and *PA16* correlated significantly with *Pseudomonas aeruginosa, P. pohangensis, P. rhodesiae*, and *P. stutzer*i. *Pseudomonas virus EL* correlated with *P. resinovorans*; *Pseudomonas virus LKA1* correlated with *P. aeruginosa, P. rhodesiae, P. stutzeri*, and *P. syringae*. *Pseudomonas virus phiCTX* correlated with *P. aeruginosa, P. pohangensis*, and *P. rhodesiae*.

The distribution of bacteria of the genera *Lactobacillus* and *Pseudomonas* is positively correlated. Moreover, the *Lactobacillus* phages and *Pseudomonas* viruses/phages are also positively correlated. *Pseudomonas resinovorans* is highly positively correlated with *Lactobacillus acidophilus*, *L. crustorum, L. iners, L. jensenii, L. kefiranofaciens, L. kullabergensis, L. plantarum*, and *L. salivarius* (r>0.80, p=0). *Pseudomonas virus EL* is correlated with *L. crustorum* and *L. iners* (r > 0.80, p = 0).

**Figure 2:**
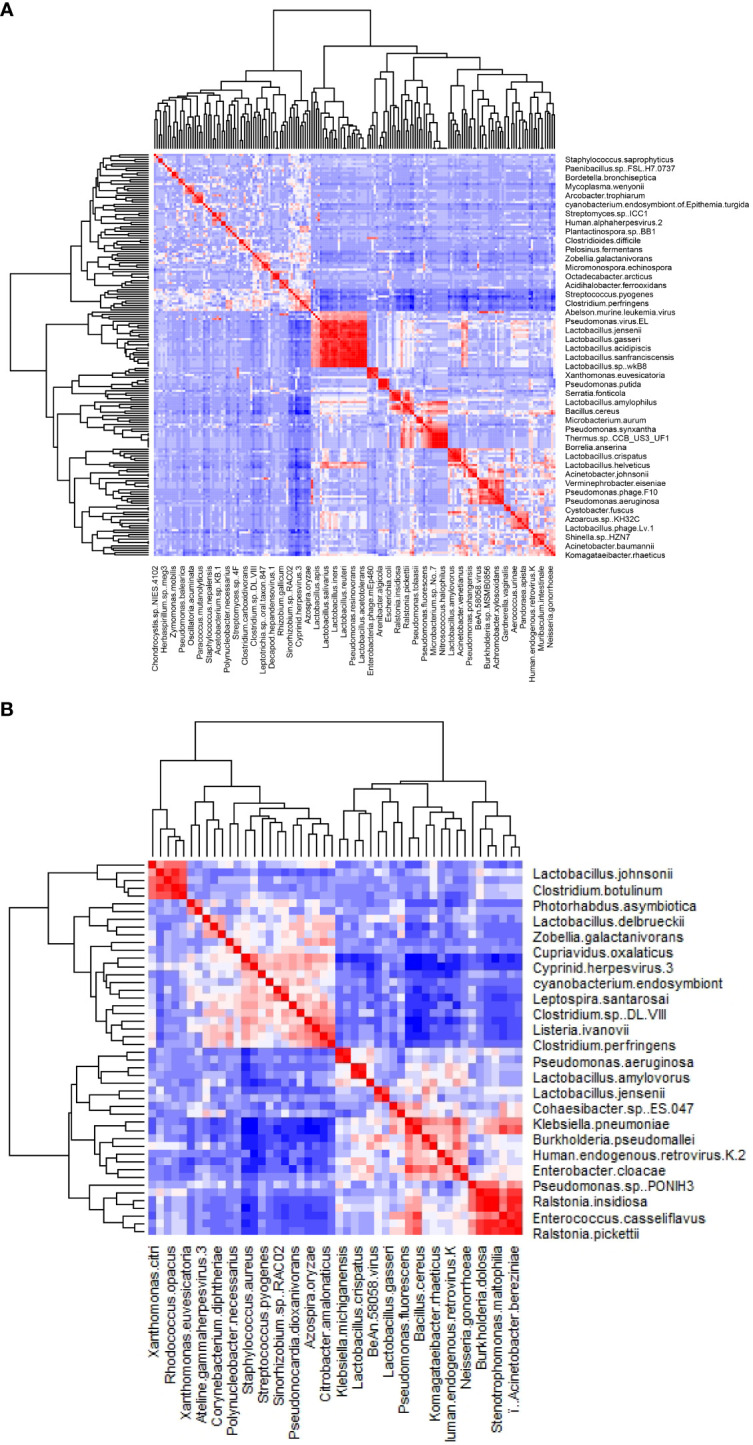
The heatmap of the correlation between detected phages/viruses and bacterial species. **(A)** All species. **(B)** Significantly correlated species with abundance above 0.1% (p<0.01).

The majority of the pathogenic bacteria had a positive correlation with each other. *Listeria ivanovii* positively correlates positively with *Clostridium perfringens*, *Clostridium sp DL VIII*, *Citrobacter amalonaticus, Azospira oryzae, Staphylococcus aureus*, and *Streptococcus pyogenes*, most of which are pathogenic. On the other hand, *L. ivanovii* negatively correlates with the benign commensals bacteria *Bacillus cereus* (**Figure 2B**). *Citrobacter amalonaticus, Corynebacterium diphtheriae, Escherichia virus IME08, Clostridium* sp. *DL VIII*, and *Listeria ivanovii* are all positively correlated with *Clostridium perfringens* (**Supplementary File 1**). The opportunistic pathogen *Staphylococcus aureus* is negatively correlated with *Lactobacillus crispatus, L. gallinarum, L. helveticus, L. kefiranofaciens, L. plantarum*, and *L. salivarius* (p<0.001).

#### 3.2.2 Viruses/phages and their correlation with bacteria

There are 24 viruses/phages annotated at the species level (**Table 2**). Among these viruses, *Cyprinid herpesvirus 3* shows the highest frequency and the widest distribution. It is detected in 33 out of the 41 samples, and the average frequency is 51.00% of the viruses/phages. *Human endogenous retrovirus K* was detected in 28 samples with an average relative frequency of 21.84%.

**Table 2:** The annotated virus/phage species and abundance.

Name	#Specimens detected out of 41	Frequency (%)				PCOS vs Non-PCOS	
		Mean		Min	Max	Ratio (PCOS/Non-PCOS)	t.test p-value
*Lactobacillus phage Lv 1*	2	0.99		0	32.58	1.32E-01	0
Pseudomonas virus EL**	9	3.19		0	35.49	6.38E-03	0
*Pseudomonas virus phiCTX*	7	0.80		0	14.37	5.15E-02	0
Pseudomonas virus LKA1*	6	0.47		0	6.54	4.17E-02	0
*Pseudomonas phage B3*	4	0.43		0	16.47	1.65E-01	0
Pseudomonas phage F10*	8	1.73		0	20.32	2.16E-02	0
Pseudomonas phage PA16**	9	1.24		0	14.07	1.73E-02	0
*Enterobacteria phage mEp460*	7	0.50		0	15.70	2.22E-01	4.78
*Microbacterium phage Min1*	4	0.01		0	0.56	1.65E-01	0
*Salmonella phage Fels 1*	4	0.10		0	3.82	1.65E-01	0
Cyprinid herpesvirus 3***	36	51.00		0	100.00	8.05E-06	3.52
Human endogenous retrovirus K***	28	21.84		0	95.61	9.95E-04	0.16
*Human alphaherpesvirus 2*	12	3.62		0	97.48	2.61E-01	2.78
*Human gammaherpesvirus 4*	4	0.03		0	1.26	1.65E-01	0
*Ateline gammaherpesvirus 3*	13	3.10		0	59.64	4.23E-01	0.80
*Alcelaphine gammaherpesvirus 1*	8	0.24		0	5.43	3.57E-01	1.64
*Abelson murine leukemia virus*	7	0.19		0	3.67	5.30E-02	0
*Callitrichine gammaherpesvirus 3*	4	0.00		0	0.13	1.65E-01	0
*Murine osteosarcoma virus*	8	2.69		0	94.85	1.84E-01	13.12
*Macacine alphaherpesvirus 1*	4	0.01		0	0.20	1.65E-01	Infinity
*Ndumu virus*	4	0.02		0	0.78	1.65E-01	Infinity
*Pestivirus Giraffe 1*	4	0.03		0	1.02	1.65E-01	0
*Ross River virus*	6	0.04		0	0.77	6.43E-02	Infinity
*Semliki Forest virus*	4	0.07		0	2.76	1.65E-01	Infinity

The relative abundance of 6 virus species (*Pseudomonas virus EL*, *Pseudomonas phage PA16*, *Pseudomonas phage F10*, *Human endogenous retrovirus K*, *Cyprinid herpesvirus 3*, and *Escherichia virus IME08*) is significantly different between groups (ANOVA test, p < 0.05). The first four of them are much more abundant in the NS (healthy control) group, while *Cyprinid herpesvirus 3* is much less abundant in the NS group (**Figure 2B**). *Cyprinid herpesvirus 3* is negatively correlated with *Pseudomonas virus EL* (r = −0.43, p = 0.0052) and *Human endogenous retrovirus K* (r = −0.55, p = 0.00016).

#### 3.2.3 Bacteria differentially enriched in PCOS and obesity groups

**Figure 3:**
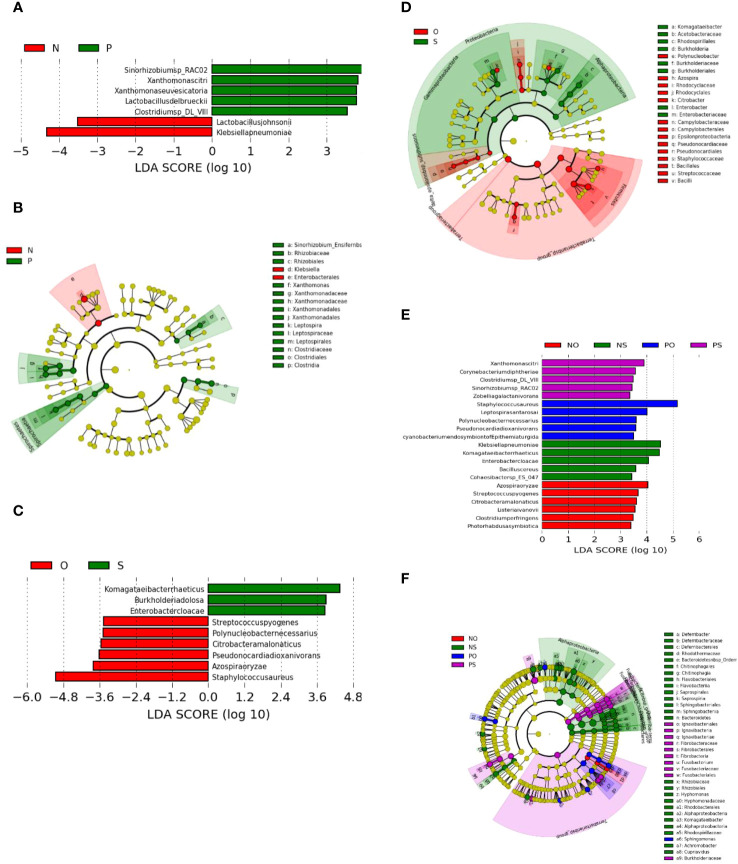
LEfSe plots of the bacteria differently enriched in the PCOS (P) group and non-PCOS (N) group, and in the Obesity (O) group and the non-obese (S) group. **(A, B)** PCOS (P) vs Non-PCOS (P); **(C, D)** Obese (O) vs Non-obese (S); **(E, F)** Non-PCOS and Obese (NO), Non-PCOS and Non-obese (NS), PCOS and Obese (PO), PCOS and Non-obese (PS).

LEfSe analysis revealed that the differentially enriched bacteria in the PCOS (P) and non-PCOS (N), and obesity (O) and non-obese (S) groups were quite different (**Figure 3**).

As shown in **Figure 3A**, the bacterial species differentially enriched in the PCOS (P) groups include *Clostridium* sp. *DL-VIII*, *Xanthomonas citri*, *Xanthomonas euvesicatoria*, *Lactobacillus delbrueckii*, and *Sinorhizobium* sp. *RAC02*. However, the bacterial species differentially enriched in the obese (O) groups are different and include *Azospira oryzae, Citrobacter amalonaticus, Polynucleobacter necessaries, Streptococcus pyogenes, Staphylococcus aureus*, and *Pseudonocardia dioxanivorans* (**Figure 3C**). Three of the enriched O-group bacteria are known as human pathogens that may be associated with the pathogenesis of obesity through inflammation (Avire et al., 2021).

The cladogram plot showed that two genera, *Xanthomonas* and *Leptospira*, and one family, *Clostridiaceae*, were enriched in the PCOS group (**Figure 3B**), while there are three genera, *Polynucleobacter, Azospira, Citrobacter*, and *Epsilonproteobacteria*, and two families, *Pseudonocardiaceae* and *Staphylococcaceae*, were enriched in the obesity group (**Figure 3D**). Bacteria of the genus *Leptospira*, which are enriched in the PCOS group, can cause a bacterial disease, leptospirosis, that affects humans and animals.

**Enriched bacteria and phages in each group were listed as follows:**

(1) Bacteria enriched only in PO, PS, NO, and NS groups:

Bacteria enriched in the NS group include *Komagataeibacter rhaeticus, Burkholderia pseudomallei, Enterobacter cloacae, Klebsiella pneumoniae, Bacillus cereus*, and *Lactobacillus.*

Bacteria enriched in the NO group include *Azospira oryzae, Clostridium perfringens, Streptococcus pyogenes, Listeria ivanovii, Citrobacter amalonaticus*, and *Photorhabdus asymbiotica.*

Bacteria enriched in the PS group include *Clostridium* sp.*DL_VIII, Xanthomonas citri, Corynebacterium diphtheriae, Zobellia galactanivorans*, and *Sinorhizobium* sp.*RAC02.*

Bacteria enriched in the PO group include *Leptospira santarosai, Polynucleobacter necessaries, Staphylococcus aureus*, and *Pseudonocardia dioxanivorans.*

(2) Bacteria and phages that are commonly enriched between the groups:

The bacteria and phages/viruses with significantly different relative abundances between PO and NS and between PS and NS are listed in **Table 3**.

**Table 3:** Bacteria and phages enrichment between PO and NS, and between PS and NS.

Name	PO_vs_NS		PS_vs_NS	
	t.test p-value	Ratio	t.test p-value	Ratio
*Staphylococcus aureus*	1.19E-05	1.85	6.65E-04	1.62
*Klebsiella michiganensis*	4.39E-02	0.22	3.13E-02	0.14
*Lactobacillus crispatus*	ND	ND	4.35E-02	0.02
*Komagataeibacter rhaeticus*	7.96E-03	0.58	ND	ND
*Klebsiella pneumoniae*	2.06E-05	0.23	9.48E-05	0.28
*Enterobacter cloacae*	1.45E-03	0.34	2.57E-02	0.54
*Xanthomonas citri*	2.41E-03	2.12	ND	ND
*Lactobacillus jensenii*	2.88E-02	0	2.88E-02	0
*Leptospira santarosai*	1.48E-03	16.78	1.54E-03	16.85
*Burkholderia pseudomallei*	1.52E-03	0.12	4.31E-04	0
*Bacillus cereus*	1.94E-04	0.07	1.88E-04	0.06
*Azospira oryzae*	5.26E-05	Infinity	1.70E-03	infinity
*Ralstonia insidiosa*	1.40E-02	0	1.40E-02	0
*Streptococcus pyogenes*	4.33E-04	3.41	ND	ND
*Staphylococcus aureus*	1.19E-05	1.85	6.65E-04	1.62
*Pseudomonas_virus_EL*	4.74E-03	0	4.74E-03	0
*Pseudomonas_virus_phiCTX*	5.16E-02	0	5.16E-02	0
*Pseudomonas_virus_LKA1*	4.13E-02	0	4.13E-02	0
*Enterobacteria_phage_mEp460*	1.04E-01	0	2.10E-01	7.17
*Lactobacillus_phage_Lv_1*	1.35E-01	0	1.35E-01	0
*Microbacterium_phage_Min1*	1.69E-01	0	1.69E-01	0
*Pseudomonas_phage_B3*	1.69E-01	0	1.69E-01	0
*Pseudomonas_phage_F10*	2.01E-02	0	2.01E-02	0
*Pseudomonas_phage_PA16*	1.57E-02	0	1.57E-02	0
*Salmonella_phage_Fels_1*	1.69E-01	0	1.69E-01	0

ND means non-detected in both groups; infinity means non-detected in the NS group.

### 3.3 Microbiota function annotation

The rarefaction curve of gene families retrieved from Humann and OTUs output from Kraken indicate that the sequencing depth is adequate for metagenomic analysis (**Supplementary Figure 3**).

The functional analysis using HUMAnN output revealed that a total of 23,438 UniRef90 genes were identified, with 1,278 classified under *Lactobacillus crispatus*, 607 under *L. iners*, and 21,553 remaining unclassified.

The pathway abundance results encompassed 27 pathways, with the majority of the samples falling within group NS and group NO. Notably, the PWY-3781 pathway, associated with aerobic respiration I (cytochrome c), was exclusively observed in the obese group. A more specific omics study should be undertaken to shed light on the functionality of the PCOS and obesity microbiome.

## 4 Discussion

The main findings of this paper are that the vaginal flora of non-PCOS and non-obese reproductive women are similar and have more bacterial phages, whereas both PCOS and obesity factors lead to alterations in the vaginal flora, and these alterations are diverse.

### 4.1 Vaginal microbiome composition and differential enrichment of bacteria

The results of this study revealed distinct differences in the composition of vaginal microbiomes between the PCOS (P) and non-PCOS (N) groups, as well as between the obesity (O) and non-obese (S) groups (**Table 3**). The non-PCOS group exhibited a relatively normal vaginal microbiome with low diversity. In contrast, the PCOS and obesity groups showed signs of dysbiosis characterized by increased microbial diversity.

Among the various bacterial genera identified in the vaginal microbiomes, several genera were significantly different in abundance between the groups. Notably, *Lactobacillus*, *Pseudomonas, Bacillus*, and other genera were enriched in the non-PCOS and non-obese (NS) group, while Staphylococcus, Clostridium, Leptospira, and others were relatively more abundant in the PCOS and obesity groups. It is worth noting that several pathogenic and opportunistic pathogens, including *Streptococcus pyogenes*, *Leptospira santarosai*, *Citrobacter amalonaticus*, *Listeria ivanovii*, and *Clostridium perfringens*, were detected in higher abundance in the PCOS and obesity groups. This finding raises concerns about potential health risks associated with the alteration of the vaginal microbiome in these conditions.

The higher abundance of benign commensals bacteria could inhibit the growth of pathogens. The fact that the pathogenic bacteria were lower in the NS group could be explained by the former finding (Kang et al., 2017); the lactic acid and other chemicals produced by *Lactobacillus* could inhibit the growth of *Staphylococcus aureus* and prevent the formation of biofilm (Gulzar, 2018). Generally, the *Lactobacillus* species is the dominant bacteria in the vaginal microbiome of healthy reproductive-age women (Spurbeck and Arvidson, 2011). *Lactobacillus* species are the common bacteria that sustain health. The abundance of *Lactobacillus* species is significantly higher in the NS group. This result is concordant with the previous study (Spurbeck and Arvidson, 2011; Greenbaum et al., 2019). *Lactobacillus* species sustain the acid and anaerobic conditions for the normal vaginal microenvironment and form the front line to defend against vaginally acquired infections (Spurbeck and Arvidson, 2011). But in the PO, PS, and NO groups, *Lactobacillus* species are not so dominant, and this suggests dysbiosis of the vaginal microbiome is linked with obesity or PCOS. The disturbance of normal vaginal bacteria has been approved to be linked with different types of diseases, such as bacterial vaginosis and virus infection (Spurbeck and Arvidson, 2011).

In this study, *Pseudomonas* species are also much higher in the NS group (**Figure 2**), suggesting they interact with each other and keep a balance. *Pseudomonas* species are not generally considered pathogenic, but studies have identified that they are present at a low level in the indigenous microbiota of various body sites, including the uterus, and can lead to pelvic inflammatory disease (Scales et al., 2014). The presence of Pseudomonas and Pseudomonas phages in the NS group but not in the PCOS or obesity groups suggests that, while Pseudomonas is a common bacteria in vaginal microbiomes.

*Bacillus cereus* is a facultative anaerobe has mechanisms for both aerobic and anaerobic respiration. Some of the major products produced from carbon sources such as sucrose or glucose during anaerobic respiration include L-lactate, acetate, formate, succinate, ethanol, and carbon dioxide (Mols et al., 2007). These metabolites produced by *B. cereus* help to sustain the acid conditions in the vaginal microenvironment and contribute to the health.

On the other hand, most of the pathogenic bacteria lower in the NS group are positively correlated with each other, such as *Listeria ivanovii*, *Clostridium perfringens*, *Citrobacter amalonaticus, Staphylococcus aureus*, and *Streptococcus pyogenes*; and *L. ivanovii* negatively correlated with the benign commensals bacteria *Bacillus cereus*. Dysbiosis can lead to the overgrowth of pathogens, which might be linked to an increased risk of infections, inflammation, and other health issues. Understanding these associations could have implications for diagnostic and therapeutic strategies in managing PCOS and obesity. This result suggested that in the PCOS or obesity groups, some groups of pathogens linked with the diseases. Furthermore, the pathogens linked with PCOS and obesity are quite different, suggesting the vaginal microbiome’s pathogenic mechanisms are different between PCOS and obesity.

In summary, the vaginal bacteria in healthy are similar, but PCOS and obesity are associated with different bacterial shifts from the normal one which are benign commensals and produce lactic acids and help to sustain the acid conditions.

### 4.2 Identification of key phages and the possible function

This study annotates 14 genera (24 species) of viruses and phages. This suggests that the distribution of viruses or phages in the vaginal microbiome is very common. Bacteriophages contribute to the maintenance of ecological balance and the evolution of bacterial species (Martín et al., 2009; Wright, 2021). Regarding the function of the vaginal bacteria phage, a previous study has illustrated that the *Lactobacillus* phage can eliminate the *Lactobacillus* culture in a petri dish (Kilic et al., 2001; Miller-Ensminger et al., 2020). But in this study, *Lactobacillus phage Lv1* was only found in two specimens of the NS group, and in these two specimens, several *Lactobacillus bacteria* were detected. This implies that the *Lactobacillus* phage contributes to the ecological balance of vaginal microecology while not eliminating the *Lactobacillus*. Furthermore, the presence of the *Lactobacillus* phage is an indicator of the presence of relatively abundant *Lactobacillus* (Miller-Ensminger et al., 2020).

*Lactobacillus phage Lv1* is a dsDNA virus isolated from a human vaginal *Lactobacillus jensenii* strain (Martín et al., 2010), which coexisted and positively correlated in this study. It has been illustrated that the stable coexistence of phages and bacteria is very common in the urogenital microbiota (Miller-Ensminger et al., 2020; Wright, 2021). The coexistence of lactobacilli and phages in the vaginal microbiome is widely spread, and many of the lysogenic lactobacilli could release phages at a high frequency (Kilic et al., 2001; Martín et al., 2009; Wright, 2021). In summary, lysogenic phages have a complex relationship with their host bacteria. They can both provide advantages and pose threats to the host, depending on the specific genes carried by the prophage and the environmental conditions. This interplay between lysogenic phages and bacteria is an important aspect of microbial ecology and evolution. lysogeny in vaginal lactobacilli is widely spread. Some lysogenic lactobacilli spontaneously release phages with a broad host range. Even though the phages were all temperate, they were able to cause lytic infection in various strains (Kiliç et al., 2001). The detailed relationship between *Lactobacillus phage Lv1 and Lactobacilli* in vaginal microbiome needs further study.

In this study, *Pseudomonas* phages/viruses, including *Pseudomonas virus EL, F10*, *phiCTX*, B*3*, PA16, and *LKA1*, have the common host *Pseudomonas aeruginosa* (Kwan et al., 2006; de Smet et al., 2017; Miller-Ensminger et al., 2020). *Pseudomonas aeruginosa* is an opportunistic pathogen that grows on the skin in moist parts, including the genital area, and it can cause severe infection when the immune system is weakened. A recent study suggested that the colonization of *Pseudomonas aeruginosa* in the genital tract can lead to infertility by inhibiting sperm mobility (Vander and Prabha, 2019). This could explain why some dysbiosis of the vaginal microbiome may lead to infertility. On the other hand, phage therapy has been proven to be an effective way to treat infection with *Pseudomonas aeruginosa* (Pires et al., 2015). The relationship between *Pseudomonas* phages/viruses and the presence of *Pseudomonas* in the vaginal microbiome, and their impacts on health needs further investigate.

The abundance of human endogenous retrovirus K (HERV-K) is significantly higher in the NS group when compared with the PS, PO, and NO groups. The most recent study suggests that HERV-K has multiple copies in the human genome and that some of its open reading frames are transcribed and translated in early embryogenesis (Garcia-Montojo et al., 2018). Though HML-2 of HERV-K has been reported to be associated with certain types of cancer and neurodegenerative diseases in adult tissues, the functional associations between HERV-K (HML-2) and diseases have not been completely clarified, and further work is still needed to confirm the specific HERV loci associated with specific diseases (Xue et al., 2020). In this study, we only sequenced DNA, not RNA, and the sequencing depth was relatively low, so it cannot reflect HERV-K transcriptional levels in the vaginal or specific HERV-K loci in the genome. The relationship between HERV-K and PCOS needs further study.

Among these viruses, *Cyprinid herpesvirus 3* showed the highest frequency and widest distribution. It is detected in 33 out of the 38 samples, and the average frequency is 50% of the viruses/phages. *Cyprinid herpesvirus 3*, also known as CyHV-3, koi herpes virus or KHV, is a species of dsDNA virus that causes a viral disease that is very contagious to the common carp *Cyprinus carpio*. CyHV-3 has been proposed as a biological control agent for the invasive carp in Australia, and the safety to non-target species has been tested, which suggests that CyHV-3 is safe for the non-target species tested, including 14 species of fish, two species each of amphibians, and reptiles, and single species of bird, mammal, and invertebrates (McColl et al., 2016). CyHV-3 is a large double-stranded DNA herpesvirus classified in the Alloherpesviridae family in the Herpesvirales order. This order encompasses herpesviruses that infect different hosts, including mammals, avians, and reptiles. CyHV-3 is divergent from the other mammalian, avian, and reptilian herpesviruses, and bears homolog genes similar to CyHV-1, CyHV-2, AngHv-1, IcHV-1, and RaHV-1 (Ilouze et al., 2012). In this study, the annotation of *Cyprinid herpesvirus 3* from the human vaginal microbiota shotgun metagenomes may be because there is a CyHV-3-like virus in the specimens. The high frequency of this CyHV-3-like virus may contribute to diseases such as PCOS. But more information about this CyHV-3-like virus in the vaginal microbiome and its function needs further study.

### 4.3 Microbial correlations

The analysis of correlations between bacterial genera and phages/viruses provided insights into the complex interactions within the vaginal microbiome. Notably, there were strong positive correlations among various *Lactobacillus* species and among *Pseudomonas* species, suggesting the presence of microbial communities with shared ecological niches. The positive correlation between *Lactobacillus phages* and *Lactobacillus*, as well as *Pseudomonas phages* and *Pseudomonas* suggest lysogenic phages are also involved in the microbiome regulation and may play an role. The third correlation is the negative one between benign commensal bacteria and pathogenic bacteria, which suggests that benign commensal bacteria play a central role in the maintenance of vaginal health. It also indicates that the disruption of the benign commensal ecology will cause a dysbiosis of the vaginal flora, which lead to invasion of pathogenic bacteria and cause PCOS in women of reproductive age by inducing inflammation, causing insulin resistance, and overproduction of androgens (Azziz et al., 2009).

### 4.4 Clinical implications

The results of this study highlight the potential clinical significance of vaginal microbiome dysbiosis in PCOS and obesity. Dysbiosis can lead to the overgrowth of pathogens, which might be linked to an increased risk of infections, inflammation, and other health issues. Understanding these associations could have implications for diagnostic and therapeutic strategies in managing PCOS and obesity. Because the interaction between the microbiota and polycystic ovary syndrome is a bidirectional one, it is likely that restoration of the vaginal flora may contribute to the relief of PCOS symptoms by reducing inflammation and restoring normal microbiome metabolites and their signaling.

### 4.5 Limitations and future directions

It’s important to acknowledge the limitations of this study, such as the relatively small sample size and the observational nature of the findings. Further research with larger and more diverse cohorts is needed to validate these results. Additionally, future studies may explore the mechanisms underlying the observed microbiome alterations and their potential impacts on gynecological and metabolic health.

## 5 Conclusion

Non-PCOS and non-obese women share a similar vaginal microbiome, but both obesity and PCOS are associated with vaginal microbiome dysbiosis with increased abundance of pathogenic bacteria. *Lactobacillus phages* and *Pseudomonas phages* are enriched in the NS group and positive correlated with their host *Lactobacilli* and *Pseudomonas*. The presence of these phages are also indicators of a healthy vaginal microbiome. The differential enrichment of bacteria in PCOS and obesity is different, suggesting that these two diseases are associated with some different microbiota mechanisms.

## Data availability statement

The datasets presented in this study can be found in online repositories. The names of the repository/repositories and accession number(s) can be found below: https://www.ncbi.nlm.nih.gov/, SRR24561304 to SRR24561344.

## Ethics statement

The studies involving humans were approved by the Human Ethics Committee of the First Affiliated Hospital of Xiamen University. The studies were conducted in accordance with the local legislation and institutional requirements. The participants provided their written informed consent to participate in this study.

## Author contributions

SZ and HY designed the project. SZ organized the data analysis and manuscript preparation. XZ, HC, TF, XQ, and MW participated in the experiments and contributed to data curation, investigation, review, and editing. All authors contributed to the article and approved the submitted version.
