# Supplementary material for: Differential enrichment of bacteria and phages in the vaginal microbiomes in PCOS and obesity: shotgun sequencing analysis
Source: Front Microbiomes. 2024 Mar 26;2:1229723. doi: 10.3389/frmbi.2023.1229723 (PMC12993533; doi:10.3389/frmbi.2023.1229723)
Supplement: Supplementary file 1 [file DataSheet_1.zip › Suppementary Tables and Figures.DOCX]

Supplementary Table 1. Participants’ BMI, blood glucose, and plasma insulin levels

| Sample ID | Group | Height（cm） | Weight（kg） | BMI | fasting plasma glucose (mmol/L) | Fasting insulin level (pmol/L) | insulin level 30 minutes after glucose administration (pmol/L) | insulin level 2 hours after glucose administration (pmol/L) |
| --- | --- | --- | --- | --- | --- | --- | --- | --- |
| ren01 | NS | 168 | 50 | 18 | 3.51 | 41.05 | 224.97 | 35.55 |
| ren10 | NS | 153 | 46 | 20 | 2.49 | 16.78 | 152.86 | 161.95 |
| ren11 | NS | 162 | 52 | 20 | 3.91 | 47.74 | 510.6 | 111.31 |
| ren12 | NS | 151 | 41 | 18 | 4.03 | 22.36 | 338.54 | 335.84 |
| ren13 | NS | 155 | 50 | 21 | 3.07 | 12.31 | 137.17 | 26.47 |
| ren14 | NS | 167 | 52 | 19 | 4.22 | 30 | 216.45 | 121.98 |
| ren15 | NS | 163 | 48 | 18 | 3.49 | 26.8 | 215.02 | 216.07 |
| ren02 | NS | 156 | 52 | 21 | 4.05 | 23.14 | 231.84 | 69.2 |
| ren03 | NS | 158 | 47 | 19 | 4.69 | 34.62 | 549.58 | / |
| ren04 | NS | 154 | 40 | 17 | 5.07 | 16.76 | / | / |
| ren06 | NS | 164 | 56 | 21 | 5.21 | 61.27 | / | / |
| ren07 | NS | 162 | 53 | 20 | 5.26 | 51.92 | / | / |
| ren08 | NS | 160 | 56 | 22 | 4.89 | 42.59 | / | / |
| ren09 | NS | 165 | 57 | 21 | / | / | / | / |
| s02 | PS | 156 | 47 | 19 | 5.88 | 69.44 | 598.05 | 551.02 |
| s03 | PS | 163 | 57 | 21 | 5.39 | 66.45 | 305.65 | 1041.7 |
| s05 | PS | 158 | 60 | 24 | 5.38 | 109.05 | 1020.87 | 562.1 |
| s06 | PS | 165 | 57 | 21 | 4.92 | 72.32 | 540.45 | 646.37 |
| s07 | PS | 165 | 48 | 17 | 1.09 | 47.23 | 187.54 | 751.75 |
| s08 | PS | 170 | 60 | 21 | 4.71 | 33.03 | / | / |
| s09 | PS | 158 | 60 | 24 | 5.03 | 82.85 | 379.05 | 1577.07 |
| s10 | PS | 144 | 48 | 23 | 5 | 92.83 | 500.25 | 801.11 |
| s11 | PO | 167 | 93 | 33 | 5.98 | 126.41 | 756.92 | 1546.17 |
| s12 | PO | 166 | 99 | 36 | 5.02 | 160.13 | 1130.44 | 1764.14 |
| s13 | PO | 155 | 82 | 34 | 5.91 | 234.54 | 956.9 | 1911.43 |
| s14 | PO | 155 | 72 | 30 | 5.46 | 112.99 | 886.98 | 726.97 |
| s15 | PO | 178 | 112 | 35 | 5.18 | 271.96 | 2496.15 | 2992.07 |
| s16 | PO | 155 | 83 | 34 | 5.54 | 74 | 366.53 | 777.49 |
| s17 | PO | 172 | 98 | 33 | 4.88 | 74.67 | 366.53 | 514.61 |
| s18 | PO | 172 | 86 | 29 | 4.65 | 243.7 | 1407.07 | 1856.84 |
| s19 | PO | 155 | 80 | 33 | 4.53 | 118.59 | 835.22 | 918.97 |
| s29 | PO | 162 | 90 | 34 | 9.13 | 171.2 | 329.87 | 744.05 |
| s30 | PO | 165 | 97 | 36 | 4.27 | 165 | 957 | 1986 |
| s20 | NO | 150 | 77 | 34 | 4.92 | 78.93 | / | / |
| s22 | NO | 163 | 76 | 29 | 5 | 57.45 | 800.04 |  |
| s23 | NO | 158 | 85 | 34 | / | / | / | / |
| s24 | NO | 155 | 90 | 37 | 7.15 | 115.59 | 179.06 | 629.35 |
| s25 | NO | 164 | 88 | 33 | 7.18 | 146.28 | 569.47 | 989.48 |
| s26 | NO | 150 | 75 | 33 | 5.47 | / | / | / |
| s27 | NO | 162 | 95 | 36 | / | / | / | / |
| s28 | NO | 157 | 78 | 32 | / | / | / | / |

Notes: NS means non-PCOS with non-obesity, PS means PCOS with non-obesity, NO means non- PCOS with obesity, PO means PCOS with obesity.


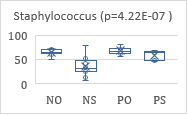

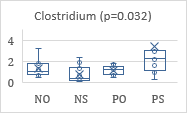

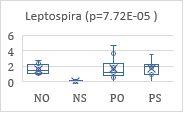

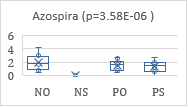

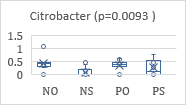

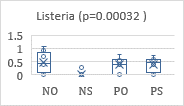

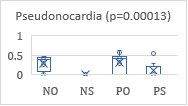

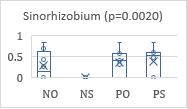

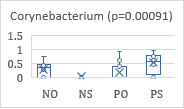

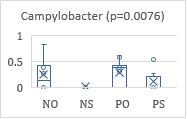

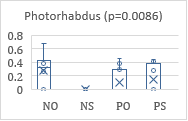

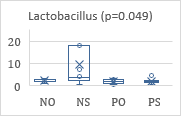

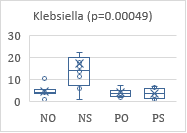

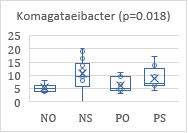

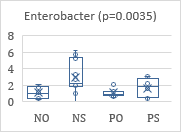

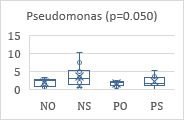

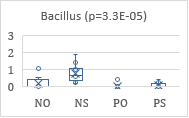

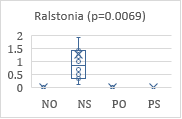

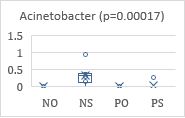

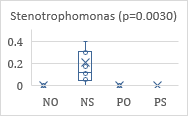

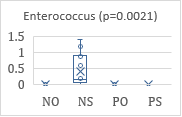


Supplementary Figure 1. the 21 bacteria genera with significantly different abundance between groups (ANOVA test, p<0.05, df between groups =3, df within groups =37).

(a)


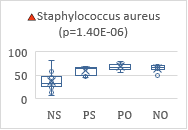

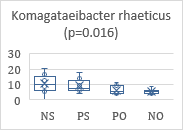

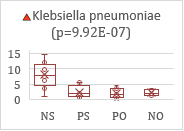

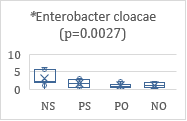

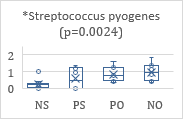

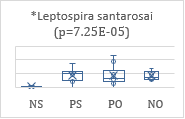

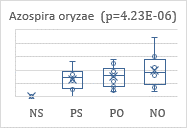

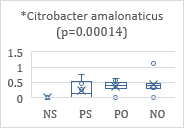

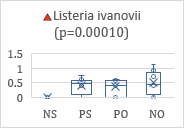

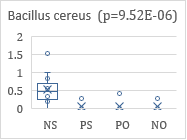

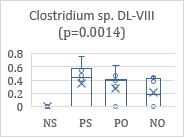

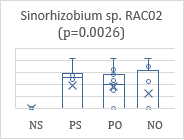

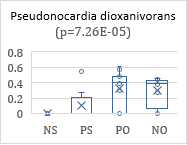

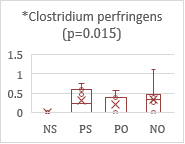

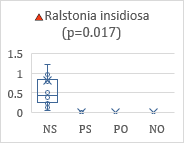

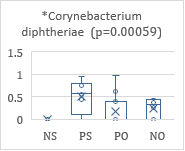

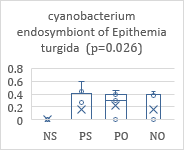

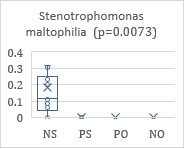

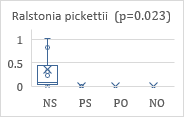

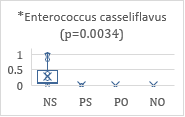

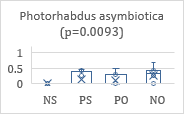

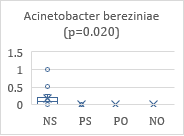

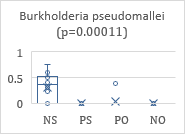

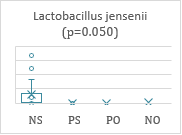

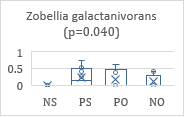

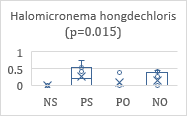


(b)


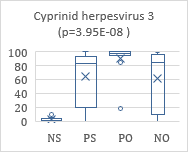

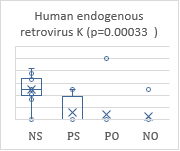

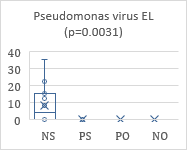

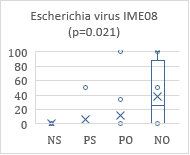

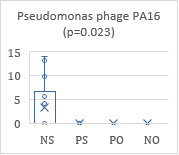

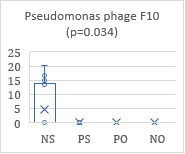


Supplementary Figure 2. the species of bacteria and phages/viruses with a significant difference in abundance between the groups (p<0.05). (a) bacteria; (b) phages/viruses

（a）


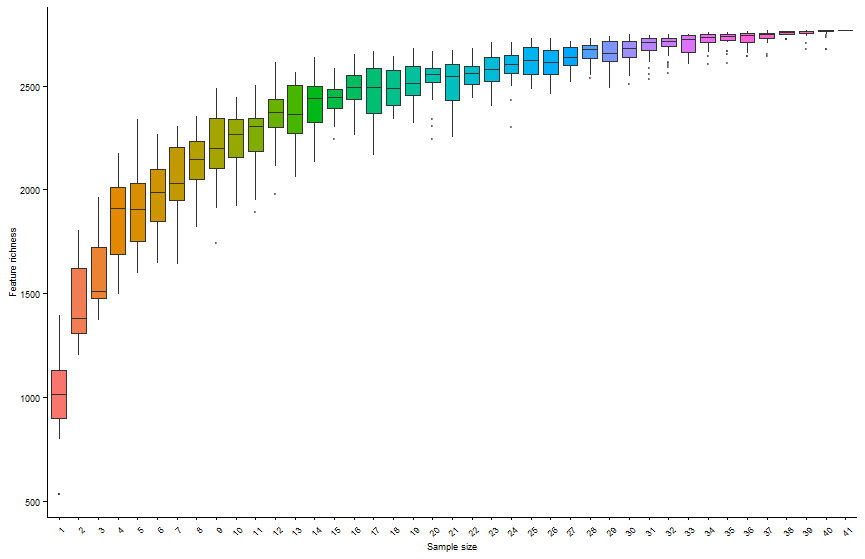


(b)


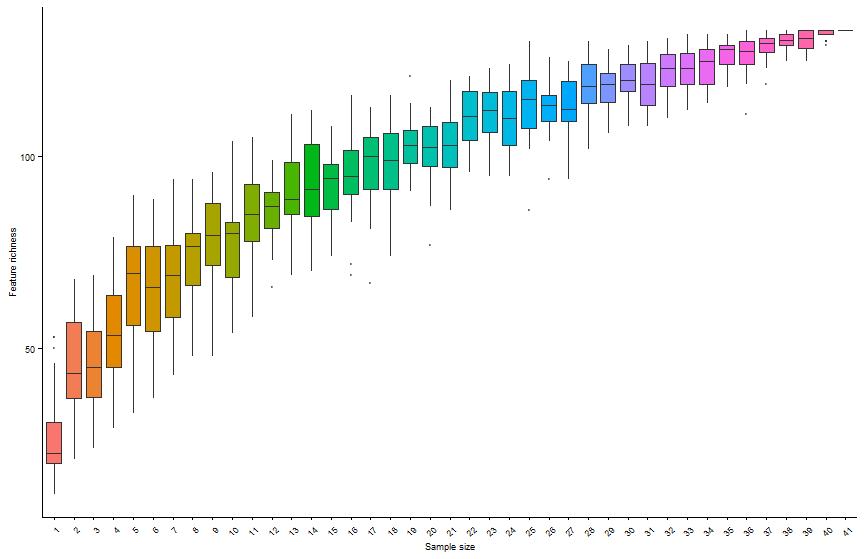


Supplementary Figure 3 The rarefaction curve of the richness of the gene families retrieved from Human. (a) rarefaction curve of gene families retrieved from Humann; (b) rarefaction curve of OTUs output from Kraken with count_cutoff=1.

Supplementary Table 2 Abundance of KEGG Pathways Annotated with HUMAnN

| Group | NS | NS | NS | NS | NS | PO | NO | NO |
| --- | --- | --- | --- | --- | --- | --- | --- | --- |
| # Pathway | ren4_ | ren10 | ren2 | ren13 | ren9 | s23 | s25 | s11 |
| GLUCOSE1PMETAB-PWY: glucose and glucose-1-phosphate degradation\|unclassified |  |  |  | 4.90 |  |  |  |  |
| LIPASYN-PWY: phospholipases\|unclassified |  |  |  | 240.96 |  |  |  |  |
| NONOXIPENT-PWY: pentose phosphate pathway (non-oxidative branch) I\|unclassified | 15.66 |  |  |  |  |  |  |  |
| P41-PWY: pyruvate fermentation to acetate and (S)-lactate I\|unclassified | 451.74 |  |  |  |  |  |  |  |
| PWY0-1061: superpathway of L-alanine biosynthesis\|unclassified | 266.44 |  |  |  |  |  |  |  |
| PWY0-862: (5Z)-dodecenoate biosynthesis I\|unclassified |  |  |  |  | 217.45 |  |  |  |
| PWY-101: photosynthesis light reactions\|unclassified |  |  |  |  |  | 31.06 |  |  |
| PWY-1269: CMP-3-deoxy-D-manno-octulosonate biosynthesis\|unclassified |  | 12.11 |  |  |  |  |  |  |
| PWY-3781: aerobic respiration I (cytochrome c)\|unclassified |  |  |  |  |  | 12.37 | 89.51 | 6.19 |
| PWY-5100: pyruvate fermentation to acetate and lactate II\|unclassified | 451.74 |  |  |  |  |  |  |  |
| PWY-6123: inosine-5'-phosphate biosynthesis I\|unclassified | 210.54 |  |  |  |  |  |  |  |
| PWY-6124: inosine-5'-phosphate biosynthesis II\|unclassified | 261.80 |  |  |  |  |  |  |  |
| PWY-6126: superpathway of adenosine nucleotides de novo biosynthesis II |  |  | 27.02 |  |  |  |  |  |
| PWY-6305: superpathway of putrescine biosynthesis\|unclassified |  | 80.96 |  |  |  |  |  |  |
| PWY-6608: guanosine nucleotides degradation III |  |  | 14.11 |  |  |  |  |  |
| PWY66-201: nicotine degradation IV\|unclassified | 2.64 |  |  |  |  |  |  |  |
| PWY-7197: pyrimidine deoxyribonucleotide phosphorylation\|unclassified |  | 31.03 |  |  |  |  |  |  |
| PWY-7208: superpathway of pyrimidine nucleobases salvage\|unclassified | 620.38 |  |  |  |  |  |  |  |
| PWY-7210: pyrimidine deoxyribonucleotides biosynthesis from CTP |  | 48.04 |  |  |  |  |  |  |
| PWY-7220: adenosine deoxyribonucleotides de novo biosynthesis II\|unclassified |  | 38.92 | 14.68 |  |  |  |  |  |
| PWY-7221: guanosine ribonucleotides de novo biosynthesis |  |  | 7.96 |  |  |  |  |  |
| PWY-7222: guanosine deoxyribonucleotides de novo biosynthesis II\|unclassified |  | 38.92 | 14.68 |  |  |  |  |  |
| PWY-7228: superpathway of guanosine nucleotides de novo biosynthesis I |  |  | 9.92 |  |  |  |  |  |
| PWY-7229: superpathway of adenosine nucleotides de novo biosynthesis I |  |  | 37.80 |  |  |  |  |  |
| PWY-7234: inosine-5'-phosphate biosynthesis III\|unclassified | 176.75 |  |  |  |  |  |  |  |
| PWY-7238: sucrose biosynthesis II\|unclassified | 859.38 |  |  |  |  |  |  |  |
| PWY-7663: gondoate biosynthesis (anaerobic)\|unclassified |  |  |  |  | 283.74 |  |  |  |
